# Supplementary material for: Magnons and magnetic fluctuations in atomically thin MnBi2Te4
Source: Nat Commun. 2022 May 9;13:2527. doi: 10.1038/s41467-022-29996-w (PMC9085848; doi:10.1038/s41467-022-29996-w)
Supplement: Supplementary file 1 — Supplementary Information [file 41467_2022_29996_MOESM1_ESM.pdf]

# Supplementary information for "Magnons and magnetic fluctuations in atomically thin layers of $MnBi_2Te_4$"

David Lujan[1,2,†], Jeongheon Choe[1,2,†], Martin Rodriguez-Vega[3*], Zhipeng Ye[4], Aritz Leonardo[5,6], T. Nathan Nunley[1,2], Liang-Juan Chang[1,7], Shang-Fan Lee[7], Jiaqiang Yan[8], Gregory A. Fiete[9,10], Rui He[4,*] & Xiaoqin Li[1,2*]

[1]*Department of Physics and Center for Complex Quantum Systems, The University of Texas at Austin, Austin, Texas, 78712, USA.*

[2]*Center for Dynamics and Control of Materials and Texas Materials Institute, The University of Texas at Austin, Austin, Texas 78712, USA*

[3]*Theoretical Division, Los Alamos National Laboratory, Los Alamos, New Mexico 87545, USA*

[4]*Department of Electrical and Computer Engineering, Texas Tech University, Lubbock, Texas, 79409, USA*

[5]*Donostia International Physics Center, Paseo Manuel de Lardizabal 4, 20018 San Sebastian, Spain*

[6]*Department of Physics, University of the Basque Country UPV/EHU, 48940, Leioa, Spain*

[7]*Institute of Physics, Academia Sinica, Taipei, 11529, Taiwan*

[8]*Materials Science and Technology Division, Oak Ridge National Laboratory, Oak Ridge, Tennessee 37831, USA*

[9]*Department of Physics, Northeastern University, Boston, Massachusetts 02115, USA*

[10]*Department of Physics, Massachusetts Institute of Technology, Cambridge, Massachusetts 02139, USA*

[†] *These authors contributed equally to this work.*

[*]*Corresponding authors:elaineli@physics.utexas.edu; rui.he@ttu.edu; rodriguezvega@lanl.gov*

**Contents**


**1 Optical microscope image and septuple layer number identification of $MnBi_2Te_4$ flakes 3**

**2 H-field dependent Raman spectra 4**

**3 Magnetic lattice and Effective spin model 5**

**4 Spin wave calculations 7**

**5 Magnon selection rules 15**

**6 First-principles calculations 27**

**7 Magnetic fluctuations in paramagnetic phase 31**

**8 Temperature dependent dynamic Raman susceptibility 33**

**9 Layer dependent low frequency phonon spectra 35**

## 1 Optical microscope image and septuple layer number identification of $MnBi_2Te_4$ flakes

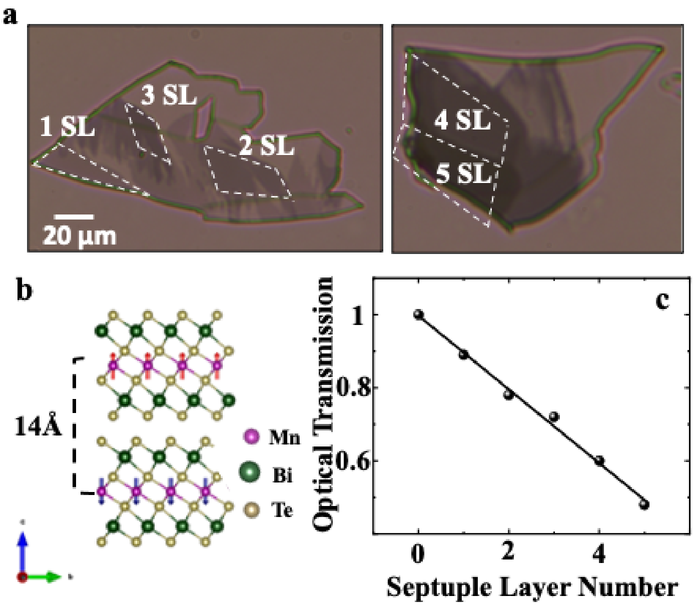


Figure S1: (a) Optical microscope images of exfoliated $MnBi_2Te_4$ flakes. The $Al_2O_3$ is deposited onto MBT flakes for exfoliation. The image was taken on a transparent PDMS substrate. Scale bar, 20 $\mu m$. (b) Illustration of the 2-SL in the AFM phase. (c) Optical transmittance (filled circles) from 1 SL to 5 SL. The solid line is a linear fit from the Beer-Lambert law.

## 2 H-field dependent Raman spectra

Systemic field dependent Raman spectra from -20 cm$^{-1}$ to 20 cm$^{-1}$ without subtracting the spin fluctuations background discuss in the main text. The left(right) panel shows spectra observed in the co-circular(cross-circular) polarization channel.

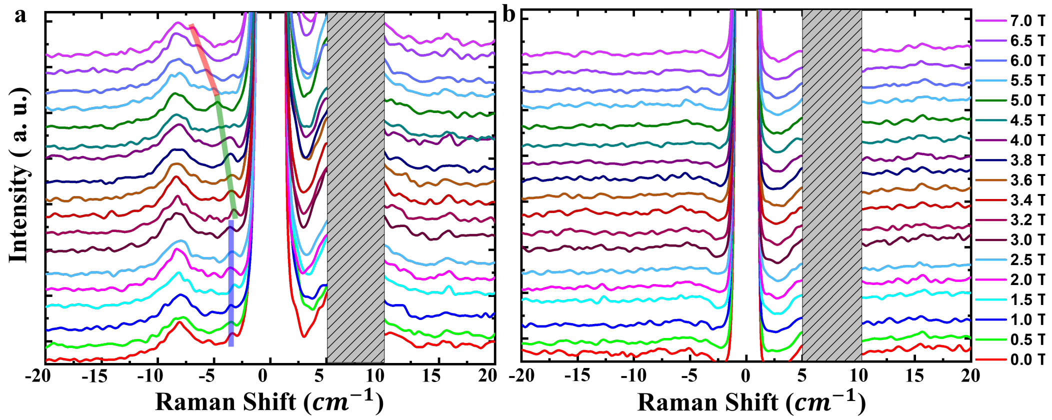


Figure S2: Magnetic field dependent Raman spectra from 2-SL MBT. (a) Low temperature (12 K) co-circular ($\sigma_+/\sigma_+$) polarization spectra. The blue, green, and red lines highlight the magnon peak in the three magnetic phases discussed in the main text. (b) Low temperature (12 K) cross-circular ($\sigma_+/\sigma_-$) polarization spectra. In both (a) and (b), the shaded gray rectangles block a noise peak that originates from the instrument.

## 3 Magnetic lattice and Effective spin model

The magnetic lattice structure for 2-SL MBT is shown in Fig. S3. In each layer, the Mn atoms form a triangular lattice in a hexagonal setting with axes $\boldsymbol{a} = A(1/2, -\sqrt{3}/2, 0)$, $\boldsymbol{b} = A(1/2, \sqrt{3}/2, 0)$, $\boldsymbol{c} = (0, 0, C)$ where $A(C)$ is the in-plane (out-of-plane) lattice constant. The Mn atoms are located at positions $\boldsymbol{r_1} = (0, 0, 0)$, $\boldsymbol{r_2} = 1/3\boldsymbol{a} + 2/3\boldsymbol{b} + \boldsymbol{c}$ in the unit cell (see Fig. S3). In each layer, the Mn atoms have six intralayer nearest neighbors (n.n.) $\delta_i$, and three interlayer n.n.s $\gamma_i$. The stacking is of the ABC type, where the top layer Mn atoms lie at the center of the triangles in the bottom layer and vice-versa, forming an effective hexagonal lattice when viewed down the $\boldsymbol{c}$-axis.

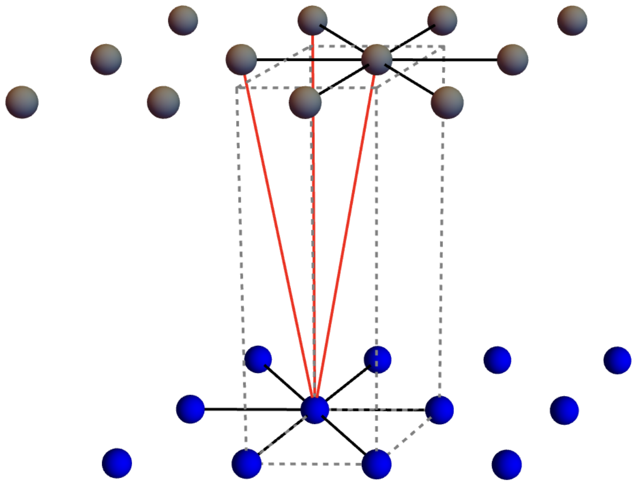

Figure S3: Bilayer MBT crystal structure, where only the Mn magnetic atoms are shown for clarity. The six (three) intralayer (interlayer) nearest-neighbors are shown by black (red) lines.

The local-moment Hamiltonian ($S = 5/2$) deduced from inelastic neutron scattering mea-

surements [1] for bulk MBT in the presence of an applied external magnetic field is

$$\mathcal{H} = -\sum_{ij} J_{ij} \boldsymbol{S}_i \cdot \boldsymbol{S}_j - J_c \sum_{\langle ij \rangle} \boldsymbol{S}_i \cdot \boldsymbol{S}_j - D \sum_i (s_i^z)^2 - g\mu_B \sum_j \boldsymbol{H} \cdot \boldsymbol{S}_i, \tag{1}$$

where $J_{ij}$ describes intralayer interactions (up to fourth-order neighbor interactions are need to correctly fit the neutron scattering data with $SJ_1 = 0.3$ meV, $SJ_2 = -0.083$ meV, and $SJ_4 = 0.023$ meV), $SJ_c = -0.055$ meV corresponds nearest-neighbor AFM interlayer interactions, and $SD = 0.12$ meV accounts for single ion anisotropy. The last term corresponds to coupling to a Zeeman field, where we assume $g = 2$ in this work and $\mu_B$ is the Bohr magneton. Ref. [2] performs inelastic neutron scattering and also finds that long-range interactions (up to seventh nearest-neighbors) are required to fit the magnon spectrum, with exchange interactions

$$[SJ_1, SJ_2, SJ_3, SJ_4] = [0.231(2), 0.033(2), 0.006(2), 0.001(2)] \text{ meV},$$

$$[SJ_5, SJ_6, SJ_7] = [0.018(2), 0.010(2), 0.001(1)] \text{ meV},$$

$SJc = 0.065$ meV, and single-ion anisotropy $SD = 0.150$ meV. This local-moment Hamiltonian and its numerical parameters lead to an A-type AFM, where each layer is FM ordered but adjacent layers present AFM order.

In this work, we adopt the model Hamiltonian Eq. (1), but restrict the intralayer interactions to nearest-neighbors. We use the interlayer exchange interaction $J_c$ and single-ion anisotropy $D$ as fitting parameters to describe our Raman scattering measurements.

## 4 Spin wave calculations

In this section, we calculate the spin wave spectrum for 2-SL in the AFM, canted AFM (cAFM), and FM phases as a function of magnetic field. Since the primitive unit cell contains two magnetic atoms in all the magnetic phases, we expect two magnon modes across all the magnetic fields considered.

**Spin order as a function of magnetic field.** 2-SL is expected to exhibit interlayer AFM order in the absence of applied magnetic field. When a magnetic field is applied in the $c$-direction, the system undergoes a first-order spin-flop phase transition into a cAFM order at the critical field $H_{sf}$ [3]. Upon further increasing the field, the system becomes FM at a second critical field $H_{fm}$. In Table 1, we summarize the reported values for the cAFM and FM critical fields for several samples.

To determine the ground state in our sample for a given magnetic field, we consider the macro-spin approximation [5], where the energy per unit volume derived from the local moment Hamiltonian (1) takes the form

$$E(\boldsymbol{M}_1, \boldsymbol{M}_2) = -\frac{Sz_1 J_1}{M_s^2}\left(|\boldsymbol{M}_1|^2 + |\boldsymbol{M}_2|^2\right) - \frac{Sz_c J_c}{M_s^2}\boldsymbol{M}_1 \cdot \boldsymbol{M}_2 - \frac{SD}{M_s^2}\left(M_{z,1}^2 + M_{z,2}^2\right) - \frac{g\mu_B}{M_s}\boldsymbol{H}_{\text{ext}} \cdot (\boldsymbol{M}_1 + \boldsymbol{M}_2), \tag{2}$$

where we assume $M_i = M_s(\sin\theta_i, 0, \cos\theta_i)$, $M_s$ is the saturation magnetization per unit cell, $z_1$ is the number of intralayer n.n.s, and $z_c$ is the number of interlayer n.n.s. in the 2-SL system. Solutions to the set of equations $\partial E(\boldsymbol{M}_1, \boldsymbol{M}_2)/\partial\theta_i = 0$ determine the possible ground states. The AFM is given by $\theta_1 = 0$ and $\theta_2 = \pi$, while the spin-flop phase by $\theta_1 = -\theta_2 = \arccos\left(-g\mu_B H_z/(2z_c S J_c + 2DS)\right)$.

**AFM magnons** When the system is in an AFM groundstate, we perform the Holstein-Primakoff transformation $\hat{S}^{+}_{a,i} = \sqrt{2S - \hat{a}^{\dagger}_{i}\hat{a}_{i}}\hat{a}_{i}$, $\hat{S}^{-}_{a,i} = \hat{a}^{\dagger}_{i}\sqrt{2S - \hat{a}^{\dagger}_{i}\hat{a}_{i}}$, $\hat{S}^{z}_{a,i} = S - \hat{a}^{\dagger}_{i}\hat{a}_{i}$, for sublattice $a$ (top layer), and $\hat{S}^{+}_{b,i} = \hat{b}^{\dagger}_{i}\sqrt{2S - \hat{b}^{\dagger}_{i}\hat{b}_{i}}$, $\hat{S}^{-}_{b,i} = \sqrt{2S - \hat{b}^{\dagger}_{i}\hat{b}_{i}}\hat{b}_{i}$, and $\hat{S}^{z}_{b,i} = -S + \hat{b}^{\dagger}_{i}\hat{b}_{i}$ for sublattice b (bottom layer). We keep terms in the transformation up to quadratic order in the bosonic operators. After Fourier transforming to momentum space we obtain the Bogoliubov–de Gennes (BdG) Hamiltonian in the basis $\psi_{\boldsymbol{k}} = (a_{\boldsymbol{k}}, b_{\boldsymbol{k}}, a^{\dagger}_{-\boldsymbol{k}}, b^{\dagger}_{-\boldsymbol{k}})^{T}$

$$H(\boldsymbol{k}) = \begin{pmatrix} A(\boldsymbol{k}) & B(\boldsymbol{k}) \\ B^{*}(-\boldsymbol{k}) & A^{*}(-\boldsymbol{k}) \end{pmatrix}, \tag{3}$$

where

$$A(\boldsymbol{k}) = \begin{pmatrix} 2DS - J_1S(f(\boldsymbol{k}) - 6) + H_z - J_cSz_c & 0 \\ 0 & 2DS - J_1S(f(\boldsymbol{k}) - 6) - H_z - J_cSz_c \end{pmatrix}, \tag{4}$$

and

$$B(\boldsymbol{k}) = \begin{pmatrix} 0 & -J_cSf_{\perp}(\boldsymbol{k}) \\ -J_cSf_{\perp}(-\boldsymbol{k}) & 0 \end{pmatrix}. \tag{5}$$

Diagonalizing $H(\boldsymbol{k})$ we obtain the magnon energies

$$\omega^{\pm}(\boldsymbol{k}) = S\sqrt{(-2D + J_1f(\boldsymbol{k}) - z_1J_1 + J_cz_c)^2 - {J_c}^2\left|f_{\perp}(-\boldsymbol{k})\right|^2} \pm g\mu_BH_z,$$

where $z_1 = 6$, $z_c = 3$, $f(\boldsymbol{k}) = \sum_i e^{i\boldsymbol{\delta}_i\cdot\boldsymbol{k}}$, $f_{\perp}(\boldsymbol{k}) = \sum_i e^{i\gamma_i\cdot\boldsymbol{k}}$ . At the $\Gamma$ point, we obtain

$$\omega^{\pm}(\boldsymbol{k} = 0) = 2S\sqrt{D(D - J_cz_c)} \pm g\mu_BH_z. \tag{6}$$

In Fig. S4, we plot the $\Gamma$ point AFM magnon energies as a function of the applied magnetic field. The corresponding eigenvectors at the $\Gamma$ point are

$$\psi^{-}_{\boldsymbol{k}=0} = 1/\mathcal{N} \begin{pmatrix} \frac{-2\sqrt{D(D-J_c z_c)}+2D-J_c z_c}{J_c z_c} \\ 0 \\ 0 \\ 1 \end{pmatrix}, \psi^{+}_{\boldsymbol{k}=0} = 1/\mathcal{N} \begin{pmatrix} 0 \\ \frac{-2\sqrt{D(D-J_c z_c)}+2D-J_c z_c}{J_c z_c} \\ 1 \\ 0 \end{pmatrix} \tag{7}$$

where $\mathcal{N}$ is a normalization constant. These wavefunctions indicate that the two sublattices oscillate with different amplitudes. This has consequence for the selection rules, as discussed in the main text.

In Fig. S5, we plot the magnon energies for $H_z = 0$ T along a high-symmetry path in the BZ. The red bands correspond to the phonons in the AFM phase obtained with density functional theory as outlined in Note 6. Notice that in the vicinity of the $\Gamma$ point, the mangnons overlap with the acoustic phonons.

**FM magnons** For the FM case, which applies for magnetic fields above the saturation field $H_{FM}$, we apply a Holstein-Primakoff transformation to the local moment Hamiltonian (1), defined by

$$\hat{S}^{+}_{a,i} = \sqrt{2S - \hat{a}^{\dagger}_i \hat{a}_i} a_i \tag{8}$$

$$\hat{S}^{-}_{a,i} = \hat{a}^{\dagger}_i \sqrt{2S - \hat{a}^{\dagger}_i \hat{a}_i}, \tag{9}$$

$$\hat{S}^{z}_{a,i} = S - \hat{a}^{\dagger}_i \hat{a}_i, \tag{10}$$

| | $H_{sf}$ | $H_{fm}$ |
|---|---|---|
| Ref. [2] (bulk) | $3.3$ T | - |
| Ref. [1] (bulk) | $3.4$ T | $7.9$ T |
| Ref. [3] (2-SL) | $2.3$ T | $3.5$T |
| Ref. [4] (4-SL) | $2.1$ T | - |
| Ref. [4] (6-SL) | $2.1$ T | $\sim 7$T |

Table 1: Magnetic field transition values reported in the literature for bulk, 2-SL, 4-SL and 6-SL MBT samples.

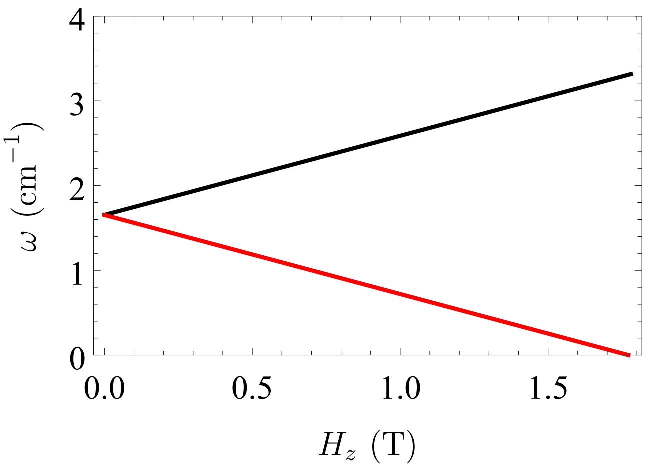


Figure S4: $\Gamma$ point AFM magnon energies as a function of the applied magnetic field. The parameters used are $SJ_c = -0.138$ meV and $SD = 0.024$ meV.

where $a$ labels the sublattice (top layer), and $i$ the atom position. The sublattice $b$ (bottom layer) has an analogous set of transformations. Keeping terms in the transformation up to quadratic order in the bosonic fields $\hat{a}_i^\dagger, \hat{a}_i, \hat{b}_i^\dagger, \hat{b}_i$, and considering only nearest-neighbors for simplicity we obtain the magnon Hamiltonian in the basis $(a_{\boldsymbol{k}}, b_{\boldsymbol{k}})$ in momentum space

$$\hat{H}_k = \begin{pmatrix} g\mu_B H_z + 2SD + SJ_c z_c - SJ_1\left(-z_1 + f(\boldsymbol{k})\right) & -SJ_c f_\perp(\boldsymbol{k}) \\ -SJ_c f_\perp(-\boldsymbol{k}) & g\mu_B H_z + 2SD + SJ_c z_c - SJ_1\left(-z_1 + f(\boldsymbol{k})\right) \end{pmatrix} \tag{11}$$

where $\boldsymbol{k} = (k_x, k_y)$ is the momentum defined in the Brillouin zone (BZ) of the triangular lattice. The magnon energies are

$$\omega_\pm(\boldsymbol{k}) = g\mu_B H_z + 2SD + z_1 SJ_1 + SJ_c z_c - SJ_1 f(\boldsymbol{k}) \pm J_c S |f_\perp(\boldsymbol{k})|. \tag{12}$$

At the $\Gamma$ point, $\omega_\pm(\Gamma) = 2SD + g\mu_B H_z + z_c S(J_c \pm J_c)$. The intralayer exchange interaction does not enter in the energy at the $\Gamma$ point. The associated wave functions are

$$(a_{\boldsymbol{k}=0}, b_{\boldsymbol{k}=0})_+ = 1/\sqrt{2}(-1, 1) \tag{13}$$

$$(a_{\boldsymbol{k}=0}, b_{\boldsymbol{k}=0})_- = 1/\sqrt{2}(1, 1). \tag{14}$$

Therefore, the $\omega_+(\Gamma)$ branch corresponds to magnetic moments in opposite layers oscillating in opposite directions, while the $\omega_-(\Gamma)$ branch corresponds to sublattices oscillating in the same direction.

In Fig. S6, we plot the magnon energies as a function of the applied magnetic field. In

Fig. S7, we plot the magnon energies along a high-symmetry path in the BZ. We also display the phonon energies (red curves).

**Canted AFM magnons** In this section we consider the magnons in the canted AFM phase (cAFM), obtained in the magnetic field regime $H_{sf} < H_z < H_{fm}$ T. The canting angle is given by $\theta_1 = -\theta_2 \equiv \theta = \arccos\left(-g\mu_B H_z/(2z_c S J_c + 2DS)\right)$. For this derivation, we follow closely Ref. [5]. We introduce a coordinate system for each layer, where the local $z$-axis is aligned with the canted moments. We apply rotations $R_y(\theta)$ and $R_y(-\theta)$ to each sublattice. Then, we apply a Holstein-Primakoff transformation to the rotated spin operators $\hat{S}^+_{m,i} = \sqrt{2S - \hat{m}_i^\dagger \hat{m}_i}\hat{m}_i$, $\hat{S}^-_{m,i} = \hat{m}_i^\dagger \sqrt{2S - \hat{m}_i^\dagger \hat{m}_i}$ , $\hat{S}^z_{m,i} = S - \hat{m}_i^\dagger \hat{m}_i$, where $\hat{m} = \hat{a}, \hat{b}$ (for the top and bottom layers). Keeping terms in the transformation up to quadratic order in the bosonic operators, going to momentum space, and applying a generalized Bogoliubov transformation, we obtain the cAFM magnon energies

$$\omega_\pm^2 = (A_k \pm D_k)^2 - (B_k \pm C_k)^2\,, \tag{15}$$

where

$$A_k = g\mu_B H_z \cos\theta + z_c S J_c \cos(2\theta) + 2SD(\cos^2\theta - \frac{1}{2}\sin^2\theta) - SJ_1(f(\boldsymbol{k}) + f^*(\boldsymbol{k}) - 2z_1) \tag{16}$$

$$B_k = -SJ_c f_\perp(\boldsymbol{k}) \sin^2\theta \tag{17}$$

$$C_k = SD\sin^2\theta \tag{18}$$

$$D_k = -SJ_c f_\perp(\boldsymbol{k}) \cos^2\theta. \tag{19}$$

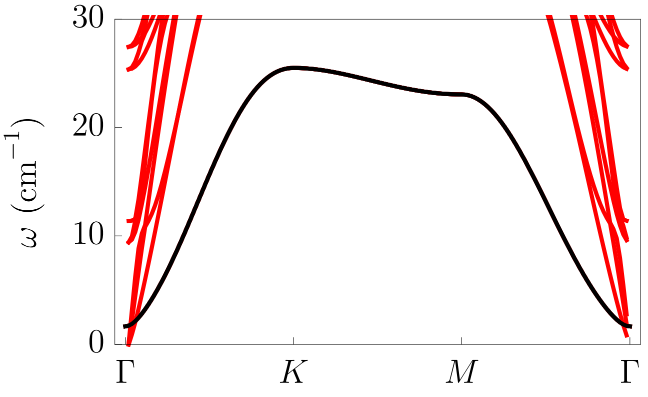


Figure S5: Magnon spectrum (black) along a high-symmetry path in the BZ for $H_z = 0$ T. The red bands correspond to phonons, as described in Note 6.

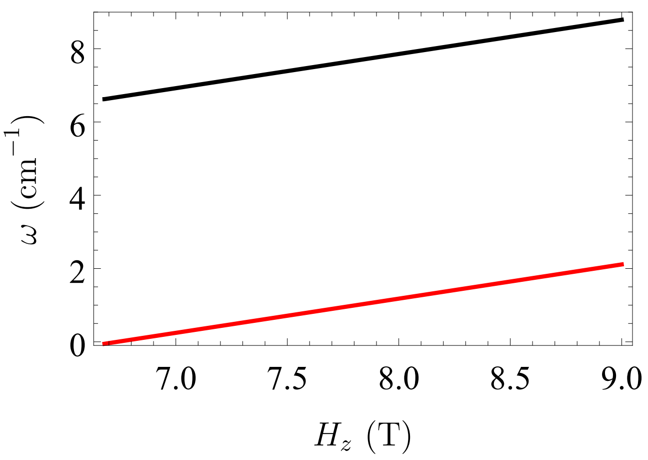


Figure S6: $\Gamma$ point FM magnon energies as a function of the applied magnetic field. Only the top branch is visible in Raman. The parameters used are $SJ_c = -0.138$ meV and $SD = 0.024$ meV.

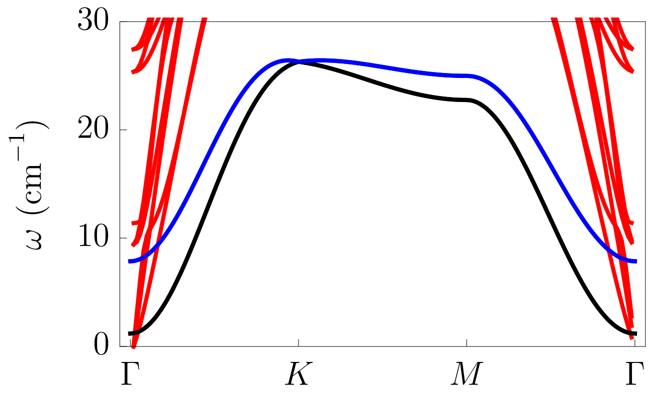


Figure S7: Magnon spectrum (black and blue) along a high-symmetry path in the BZ for $H_z = 8$ T. The red bands correspond to phonons. The parameters used are $SJ_c = -0.138$ meV and $SD = 0.024$ meV.

At the $\Gamma$ point, the cAFM magnon energies simplify to

$$\omega_+ = \sqrt{SJ_c z_c \left( (g\mu_B H_z)^2 \frac{(SJ_c z_c - SD)}{(SJ_c z_c + SD)^2} + 4DS \right)} \tag{20}$$

$$\omega_- = 0. \tag{21}$$

In Fig. S8, we plot the cAFM magnon energies as a function of the magnetic field.

## 5 Magnon selection rules

In this section, we discuss the selection rules for the magnon modes in 2-SL MBT in each of the three ground states as a function of magnetic field. In analogy with the selection rule derivation for phonons, we need to take into account the magnetic point group and analyze the symmetries of the magnetic moment oscillations. For our symmetry analysis, we employ the ISOTROPY Software Suite [6,7], and the Bilbao Crystallographic Server [8].

In the paramagnetic phase, 2-SL MBT belongs to the space group $P\bar{3}m1$ with point group $D_{3d}$, with $D_{3d} = D_3 + iD_3$ and $D_3 = \{\mathbb{1}, 3_z, 3_z^{-1}, 2_x, 2_{xy}, 2_y\}$. We can understand this difference in space group compared with bulk MBT because 2-SL MBT (and N-SL in general) loses the symmetries composed with translations along the $c$ axis. In 2-SL MBT, neither FM nor AFM order changes the size of the chemical unit cell, and the wave vector for the magnetic order is $\boldsymbol{k} = (0, 0, 0)$. This is relevant to derive the possible symmetry-allowed magnetic orders. For an extended discussion, see Ref. [9]. In the next sections, we obtain the magnetic point group in each phase, the corresponding Raman tensors, and the expected one-magnon selection rules. We then study two-magnon Raman processes, and the effect of broken inversion symmetry.

**AFM phase** AFM 2-SL MBT possesses magnetic space group $P\bar{3}'m'1$, as determined by ISOTROPY [6,7]. The corresponding magnetic point group is $D_{3d}(D_3) = D_3 \oplus \mathcal{T}(iD_3)$ ($\bar{3}'m'$), where $\mathcal{T}$ is the time reversal operator, $i$ corresponds to inversion, and the point group $D_3 = \{\mathbb{1}, 3_z, 3_z^{-1}, 2_x, 2_{xy}, 2_y\}$ is the corresponding unitary invariant subgroup of index two. This is relevant to obtain the magnon Raman tensors we discuss below.

Notice that neither time reversal $\mathcal{T}$ nor inversion $i$ are symmetries of the magnetic point group $D_{3d}(D_3)$ ($\bar{3}'m'$). The magnetic point groups possess irreducible corepresentations, in analogy with point groups and their irreducible representations. In analogy with phonons, magnons at each wave vector can be classified according to each of the magnetic point group corepresentations, which is relevant to determine their selection rules. The character tables of all 122 magnetic point groups can be found in the Bilbao Crystallographic Server [10–12], which we use to determine the magnon corepresentations considering their wave functions.

For $D_{3d}(D_3)$ ($\bar{3}'m'$), the characters of the unitary symmetry operations are shown in Table 2.

According to the group theory of magnetic point groups, the selection rules for the $\Gamma$ point optical magnons are dictated by the unitary invariant subgroup of the magnetic point group [13]. AFM 2-SL MBT has unitary invariant subgroup $D_3$, which does not include inversion symmetry. Therefore, the usual cross-circular polarization selection rule does not apply in this phase. The Raman tensors for $D_{3d}(D_3)$ are given in terms of the corepresentations by [14]

$$R(DA_1) = \begin{pmatrix} a & 0 & 0 \\ 0 & a & 0 \\ 0 & 0 & b \end{pmatrix}, R(DA_2) = \begin{pmatrix} 0 & b & 0 \\ -b & 0 & 0 \\ 0 & 0 & 0 \end{pmatrix},$$

$$R^{(1)}(DE) = \begin{pmatrix} c & 0 & 0 \\ 0 & -c & d \\ 0 & d & 0 \end{pmatrix}, R^{(2)}(DE) = \begin{pmatrix} 0 & -c & -d \\ -c & 0 & 0 \\ -d & 0 & 0 \end{pmatrix}, \tag{22}$$

where all the coefficients are real. The Raman intensity is then given by $I_\nu \propto \left| \sum_{\alpha,\beta=\{x,y,z\}} e_{\text{in}}^{*\,\alpha} R^{\nu}_{\alpha\beta} e^{\beta}_{\text{out}} \right|^2$, where $e^{\alpha}_{\text{in/out}}$ are the polarization vectors of the incoming and outgoing light and $R^{\nu}_{\alpha\beta}$ are the com-

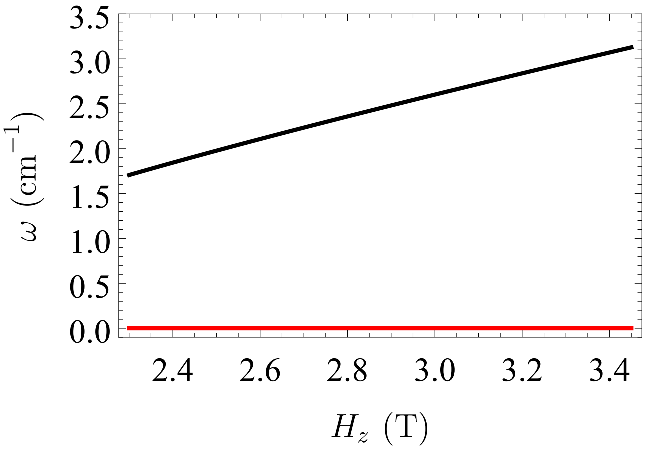


Figure S8: $\Gamma$ point cAFM magnon energies as a function of the applied magnetic field. Only one of the magnon modes has non-zero energy. The parameters used are $SJ_c = -0.138$ meV and $SD = 0.024$ meV.

| | $\mathbb{1}$ | $3_z, 3_z^{-1}$ | $2_x, 2_{xy}, 2_y$ |
|---|---|---|---|
| $\mathrm{DA}_1$ | 1 | 1 | 1 |
| $\mathrm{DA}_2$ | 1 | 1 | $-1$ |
| DE | 2 | $-1$ | 0 |

Table 2: The characters of the unitary symmetry operations of the magnetic point group $D_{3d}(D_3)$ ($\bar{3}'m'$).

ponents of the Raman tensor for mode $\nu$ [15]. Then, a magnon with symmetry $DA_1$ can be observed in the $\sigma_+/\sigma_+$ channel, and magnons with symmetry $DE$ can be observed in the $\sigma_+/\sigma_-$ channel.

The corepresentation of the magnons in AFM 2SL-MBT can be obtained by studying the transformation properties of the magnon wave functions derived in the previous sections [16]. At the $\Gamma$ point, we find $|\psi^-(\boldsymbol{k}=0)\rangle = 1/\mathcal{N}\left(\mathcal{C}\hat{a}+\hat{b}^\dagger\right)$ and $|\psi^+(\boldsymbol{k}=0)\rangle = 1/\mathcal{N}\left(\mathcal{C}\hat{b}+\hat{a}^\dagger\right)$, where $\mathcal{C}$ is a coefficient that depends on the interlayer exchange and anisotropy (see Eqns. (7)).

We can simplify the analysis by studying the transformation under rotations $\{2_x, 2_{xy}, 2_y\}$, which switch the sublattices (top and bottom layers). Since the coefficients of the operators $\hat{a}, \hat{b}^\dagger$ are different (this is, magnetic moments in opposite layers oscillate with different amplitudes), the symmetries $\{2_x, 2_{xy}, 2_y\}$ are lost and the AFM magnons should transform as the doubly-degenerate $DE$ corepresentation. The Raman tensors $R(DE)$ indicate that this modes are observable in the cross-polarization channel.

**FM phase** FM 2-SL MBT has magnetic space group $P\bar{3}m'$, as determined by ISOTROPY [6,7]. The corresponding magnetic point group is $D_{3d}(S_6) = S_6 + \mathcal{T}(D_{3d} - S_6)$ ($\bar{3}m'$), where $S_6 = \{\mathbb{1}, 3_z, 3_z^{-1}, i, i3_z, i3_z^{-1}\}$ is the unitary invariant subgroup. The magnon selection rules are determined by the unitary invariant subgroup $S_6$, which possess inversion symmetry, contrary to the

AFM case. The Raman tensors for the magnetic point group corepresentations are [14]

$$R(DA_g) = \begin{pmatrix} a & ib & 0 \\ -ib & a & 0 \\ 0 & 0 & l \end{pmatrix}, R(DE_g^{(1)}) = \begin{pmatrix} d & id & ic \\ id & -d & c \\ ig & g & 0 \end{pmatrix}, R(DE_g^{(2)}) = \begin{pmatrix} e & -ie & -if \\ -ie & -e & f \\ -ih & h & 0 \end{pmatrix}. \tag{23}$$

At the $\Gamma$ point, we find the magnon creation operators $|\psi^{\pm}(\boldsymbol{k}=0)\rangle = \left(\mp\hat{a}+\hat{b}\right)/\sqrt{2}$ (see Eqns. (14)). The simplest symmetry to analyze in this case is inversion, which switches sublattices and does not affect the magnetic moment direction: $i|\psi^{\pm}(\boldsymbol{k}=0)\rangle = \mp|\psi^{\pm}(\boldsymbol{k}=0)\rangle$. Therefore, $|\psi^{+}(\boldsymbol{k}=0)\rangle$ is Raman silent and $|\psi^{-}(\boldsymbol{k}=0)\rangle$ is Raman active with energy $\omega_{\pm}(\Gamma) = 2SD + g\mu_B H_z + z_c S(J_c \pm J_c)$. Since $|\psi^{-}(\boldsymbol{k}=0)\rangle$ is also invariant under rotations $3_z, 3_z^{-1}$, and rotations combined with inversion $i3_z, i3_z^{-1}$, we deduce it transforms as the corepresentation $DA_g$, which can be observed in the $\sigma_+/\sigma_+$ polarization channel.

**cAFM phase** In the cAFM phase, the magnetic space group is $C2'$, as determined by ISOTROPY [6,7]. The corresponding not centro-symmetric magnetic point group $2' = \{\mathbb{1}, \mathcal{T}2_y\}$. The Raman tensor for the magnetic point group $2'$ corepresentations is [14]

$$R(DA) = \begin{pmatrix} a & b & ic \\ d & e & if \\ ig & ih & l \end{pmatrix}. \tag{24}$$

In this magnetic phase, due to the low-symmetry, the Raman tensor components do not have many restrictions, and a magnon could be observed in the $\sigma_+/\sigma_+$ and $\sigma_+/\sigma_-$ polarization channels,

depending on the intensity of the matrix elements. These intensities cannot be obtained within group theory and require first principles calculations.

**Spin wave Raman tensors** We list the Raman elements of all possible observable modes in different magnetic phases in MBT 2-SLs. Experimentally, not all modes allowed by the Raman tensor are observed. One needs to compute the magnon wavefunctions for a particular material using spin wave theory. The calculated magnon modes obey a certain symmetry (e.g. $DA_1$, $DE$) that further predicts the polarization configurations of the Raman signal.

| Magnetic Phase | Raman Tensor | $\sigma_+/\sigma_+$ ($\sigma_-/\sigma_-$) | $\sigma_+/\sigma_-$ ($\sigma_-/\sigma_+$) |
|---|---|---|---|
| AFM | $DE = \begin{pmatrix} c & 0 & 0 \\ 0 & -c & d \\ 0 & d & 0 \end{pmatrix}$ or $\begin{pmatrix} 0 & -c & -d \\ -c & 0 & 0 \\ -d & 0 & 0 \end{pmatrix}$ | 0(0) | $4\|c\|^2$ $(4\|c\|^2)$ |
| | $DA_1 = \begin{pmatrix} a & 0 & 0 \\ 0 & a & 0 \\ 0 & 0 & b \end{pmatrix}$ | $4\|a\|^2(4\|a\|^2)$ | 0(0) |
| | $DA_2 = \begin{pmatrix} 0 & b & 0 \\ -b & 0 & 0 \\ 0 & 0 & 0 \end{pmatrix}$ | 0(0) | $4\|b\|^2(4\|b\|^2)$ |
| c-AFM | *DA* = $\begin{pmatrix} a & b & ic \\ d & e & if \\ ig & ih & l \end{pmatrix}$ | $(a+e)^2+(d-b)^2$ $((a+e)^2+(d-b)^2)$ | $(a-e)^2+(d+b)^2$ $((a-e)^2+(d+b)^2)$ |
| FM | $DA_g = \begin{pmatrix} a & ib & 0 \\ -ib & a & 0 \\ 0 & 0 & l \end{pmatrix}$ | $4\|a+b\|^2(4\|a-b\|^2)$ | 0(0) |
| | $DE_g = \begin{pmatrix} d & id & ic \\ id & -d & c \\ ig & g & 0 \end{pmatrix}$ or $\begin{pmatrix} e & -ie & -if \\ -ie & -e & f \\ -ih & h & 0 \end{pmatrix}$ | 0(0) | $4\|d\|^2(4\|e\|^2)$ |

Table 3: Column (ii) Raman tensors for a certain magnon mode with a specific symmetry (e.g., *DE* or *DA*); Tensor elements contributing to the Raman signal with (ii) co-circular and (iii) cross-circular configurations. Those elements responsible for the observed magnon mode discussed in the main text are highlighted in red. The selection rule is valid for one magnon scattering processes.

**Symmetry Operations** The c-AFM phase belongs to the 2' point group where two-fold rotational symmetry along the y-axis ( $\mathcal{C}_y$) is broken. On the other hand, in the AFM case two-fold rotational symmetry along the xy plane ($\mathcal{C}_y$,$\mathcal{C}_{yx}$,$\mathcal{C}_x$) are lost. So in the c-AFM case, one must rotate along the y-axis while the AFM case can be either x or y-axis.

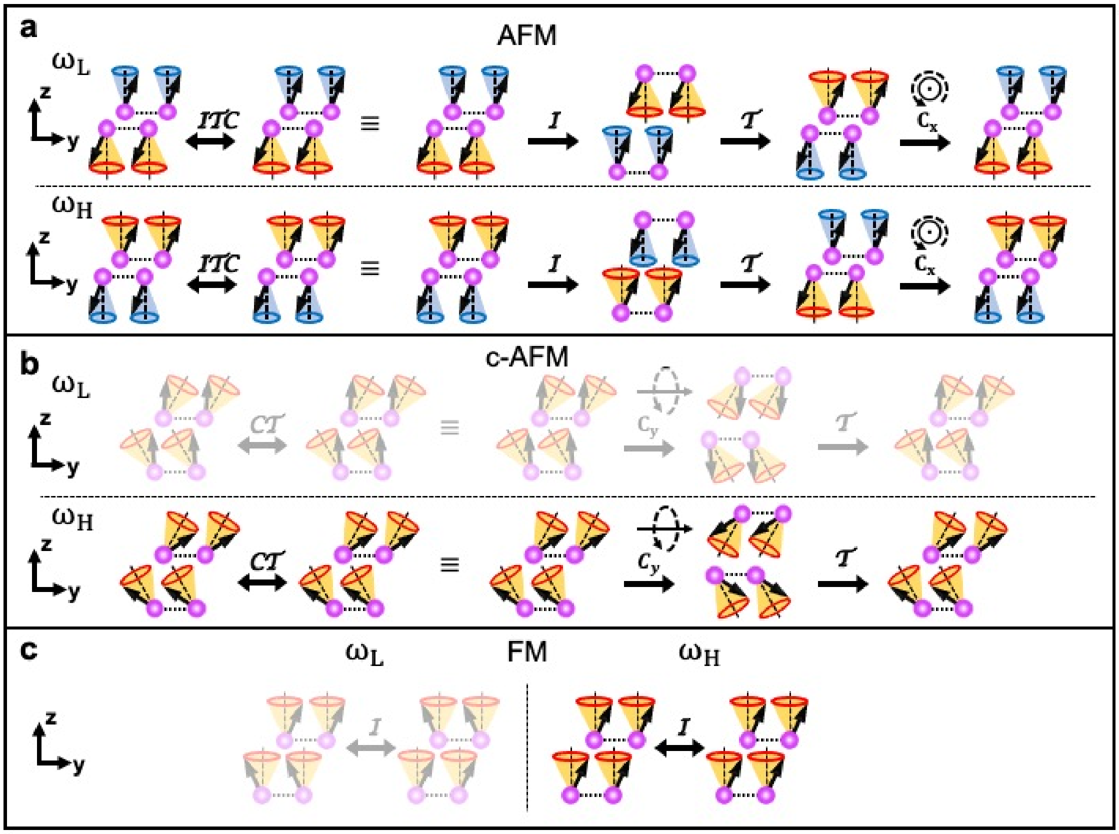


Figure S9: (a - c) Illustration of the step by step symmetry operations for each magnetic phase of 2-SL MBT.

In summary, even though the symmetries are different in each of the magnetic ground states due to the direction of the magnetic moments, one-magnon processes can be observed in the $\sigma_+/\sigma_+$ polarization channel for cAFM and FM phases. In the AFM phase, the one-magnon processes are

expected in the cross-polarization channel $\sigma_+/\sigma_-$.

**Two-magnon Raman scattering** In this section, we consider the possibility that the magnetic mode at zero applied magnetic field originates from two-magnon Raman scattering processes.

There are two features that support this interpretation. First, $M_1$ integrated intensity decreases as a function of temperature and vanishes at $T_c \approx 17K$, consistent with the critical temperature for 2-SL MBT [3]. Second, the energy scale of this mode is consistent with interlayer exchange $J_c$ and anisotropy $D$ interactions we obtained by fitting the cAFM magnons. However, we should point out that its linewidth is relatively small compared with other two-magnon processes reported in the literature [17–20].

In Fig. S10 (left), we plot the AFM magnons at zero applied magnetic field considering only nearest-neighbour intralayer interactions along a high symmetry path in the Brillouin zone. The two magnon modes are degenerate at zero magnetic field. At the $\Gamma$ point, the magnons are gaped out due to the finite single-ion anisotropy $D$. Notice that for 2-SL MBT, the chemical and magnetic unit cells are identical. In the density of states $\rho(\omega)$, Fig. S10 (right), this gap manifests as a step-like increase which contributes to two-magnon processes, since it determines the distribution of scattering intensity.

To model two-magnon Raman scattering processes, we consider the Loudon-Fleury scattering operator for spin-zero excitations [21–24]

$$\mathcal{O}_{\text{LF}} = \sum_{\langle i,j \rangle} J_{ij} \left(\hat{e}_{\text{in}} \cdot \hat{d}_{ij}\right) \left(\hat{e}_{\text{out}} \cdot \hat{d}_{ij}\right) \vec{S}_i \cdot \vec{S}_j, \tag{25}$$

where $J_{ij}$ are the exchange constants, $\hat{d}_{ij}$ are the unit vectors between sites $i$ and $j$ belonging

to opposite sublattices (in our case, opposite layers), and $\hat{e}^{\text{in(out)}}$ is the incoming (outgoing) electric field polarization. In the $\sigma_+/\sigma_+$ channel, we find that the Raman intensity [22,25,26] $I(\omega) = \sum_{\mu} |\langle\mu|\mathcal{O}_{\text{LF}}|0\rangle|^2 \delta\left(\omega - \omega_\mu + \omega_0\right)$, where $|0\rangle$ is the ground state with energy $\omega_0$, $\{|\mu\rangle, \omega_\mu\}$ are the two-magnon energy and eigenstates obtained from the spin-wave Hamiltonian in the non-interacting limit. We find that the two-magnon Raman intensity $I(\omega)$ captures all the characteristics of the density of states, as shown in Figure S11, where we consider the interlayer exchange interaction in the Loudon-Fleury scattering operator. Since this is a spin zero excitation, it has no magnetic field dependence.

Therefore, the absence of magnetic field dependence of a zero-spin excitation and the energy scale support the hypothesis that the magnetic mode at zero and low-applied magnetic field can be associated to a two-magnon process.

We note that in $LiMnPO_4$, an antiferromagnetic material with a spin-flop transition at $\sim 4$ T [26], the two-magnon responses are observed for magnetic fields above the transition field [25].

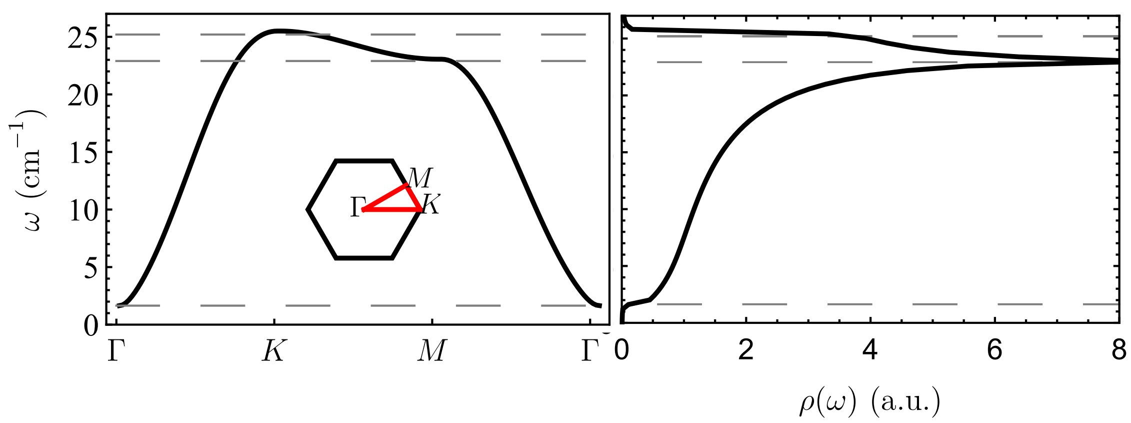


Figure S10: (Left) AFM magnon energies along a high-symmetry path in the BZ as indicated in the inset. (Right) Magnon density of states as a function of energy. The gray dashed lines link the magnon band structure with the density of states step-like and peak features.

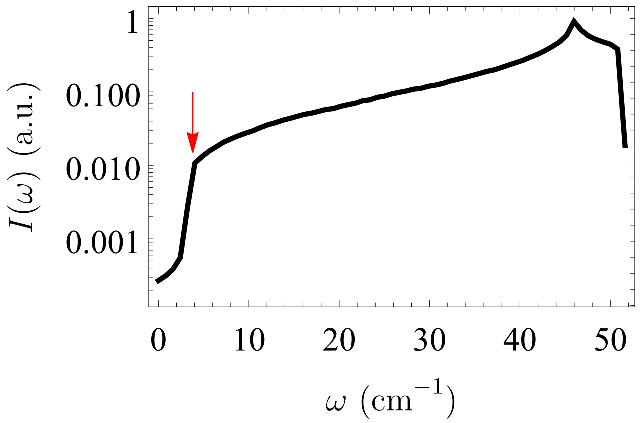


Figure S11: Two magnon Raman intensity as a function of energy in the $\sigma_+/\sigma_+$ channel. The red arrow indicates the step-like feature possibly associated with experimental observation.

**Effect of broken inversion symmetry on the Raman selection rules** In the paramagnetic phase, the point group of the crystal is $D_{3d}$, which possess inversion symmetry. When inversion symmetry is broken, for example as the result of strong coupling with the substrate, the point group reduces to $C_{3v}$. The correlation relation table is [8]

| $D_{3\text{ d}}(-3\text{ m})$ | $C_{3\text{v}}(3\text{ m})$ |
|---|---|
| $\text{A}_{1\text{ g}}$ | $A_1$ (Raman active) |
| $A_{1\text{u}}$ | $A_2$ |
| $A_{2\text{ g}}$ | $\text{A}_2$ |
| $A_{2\text{u}}$ | $\text{A}_1$ (Raman active) |
| $E_{\text{g}}$ | $E$ (Raman active) |
| $\text{E}_{\text{u}}$ | $E$ (Raman active) |

(26)

Infrared modes (such as $E_u$, $A_{2u}$) can become Raman active. From the above table, the modes with frequency $3.6$ cm$^{-1}$ observed in the $\sigma_+/\sigma_+$ channel at zero field could originate from an $A_{2u}$ mode. Our DFT calculations (see Sec. 6) indicate that the lowest $A_{2u}$ mode has frequency $38.35$ cm$^{-1}$. We should note that the absence of magnetic field dependence in this $A_{2u}$ phonon is expected from the morphic effect point of view. General group theory results indicate that non-degenerate phonons (such as $A$-type modes) cannot acquire a magnetic field dependent frequency [27]. On the other hand, 2-SL MBT $E$-type modes can acquire a magnetic field-dependent frequency of the form $\omega(H_z) \approx \omega(H_z = 0) \pm \kappa H_z/\omega(H_z = 0)$ [27].

We note that below the transition temperature AFM order sets in, which also breaks inversion

symmetry, and can also change the phonon selection rules.

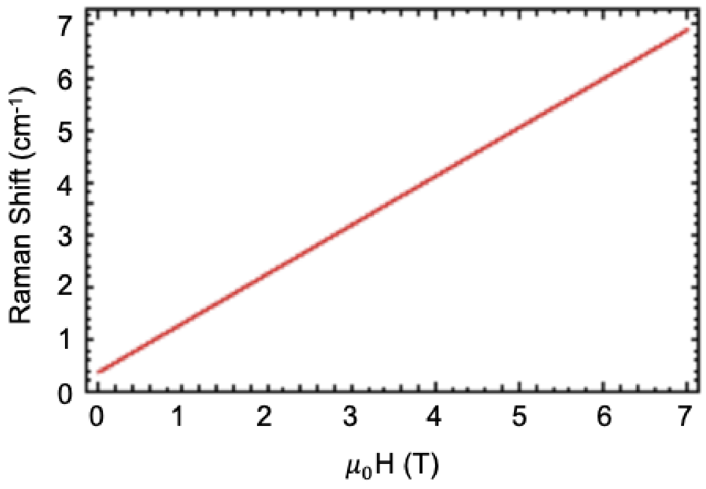


Figure S12: Monolayer Magnon at the Gamma point

**Monolayer Magnon at the Gamma point** Theoretical calculation of the monolayer(1-SL) magnon as a function of external magnetic field, fig.S12. Our limit of detection is 3.5 $cm^{-1}$ which the magnon is not observed at the ground state. At 6T the mode should be above our detection range with an energy of 6 $cm^{-1}$. In the case of the 1-SL, the magnetic anisotropy could be weak were the magnon may not be as rebust as that of the 2-SL.

## 6 First-principles calculations

**Electronic spectrum** In this section, we calculate the electronic band structure for AFM 2-SL MBT by taking into account spin-orbit coupling. We find an energy gap of approximately 80 meV much larger than the observed mode. We use the DFT package VASP [28,29], the generalized gradient

approximation (GGA) with PAW potentials with an energy cut-off of 700 eV, a $11 \times 11 \times 1$ k-point Monkhorst pack mesh, gaussian smearing with 0.01 eV width, van der Waals corrections DFT-D3 method of Grimme [30]. The parameter $U = 5.3$ eV is introduced in the Mn atoms, following the literature [31]. Here, we perform a non-collinear calculation in the AFM configuration with SOC included. To describe the bilayer we have introduced a $16$ Å vacuum between bilayers.

**Phonon spectrum** Lattice-dynamics calculations were performed using the supercell finite-displacement method implemented in the Phonopy software package [32], with VASP [28,29] used as the 2nd order force-constant calculator. Calculations of the phonon supercell size were carried out on $2 \times 2 \times 1$ expansions of the primitive-cell. For the DFT force calculations we employ the same setup as described for the electronic band structure.

The phonon spectrum is presented in Fig. S14. In Table 6, we show the phonons at the $\Gamma$ point, along with the irreps.

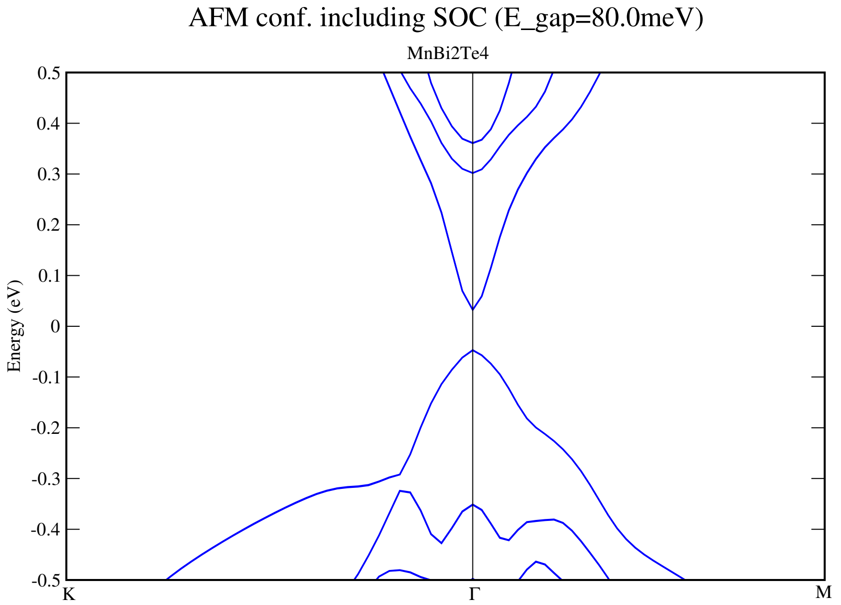


Figure S13: Electronic band structure along a high-symmetry path in the BZ. We take into account spin-orbit coupling in this calculation.

| Frequency (THz) | Irrep |
|---|---|
| 0.00 | $E_u$ |
| 0.00 | $A_{2u}$ |
| 0.294211 | $E_g$ |
| 0.388741 | $A_{1g}$ |
| 0.742719 | $E_u$ |
| 0.808097 | $E_g$ |
| 1.15487 | $A_{2u}$ |
| 1.26773 | $A_{1g}$ |
| 1.60084 | $E_u$ |
| 1.66799 | $E_g$ |
| 1.91658 | $E_u$ |
| 2.06342 | $E_g$ |
| 2.61844 | $A_{2u}$ |
| 2.63803 | $A_{1g}$ |
| 2.70516 | $E_u$ |
| 2.81849 | $E_g$ |
| 3.01212 | $E_u$ |
| 3.10706 | $E_g$ |
| 3.45274 | $A_{1g}$ |
| 3.46327 | $A_{2u}$ |
| 3.90221 | $E_u$ |
| 3.91189 | $E_g$ |
| 4.24963 | $A_{1g}$ |
| 4.26792 | $A_{2u}$ |
| 4.37695 | $A_{1g}$ |
| 4.37978 | $A_{2u}$ |
| 4.69355 | $A_{2u}$ |
| 4.72188 | $A_{1g}$ |

Table 4: $\Gamma$ point phonons and their irreducible representation obtained from first-principles.

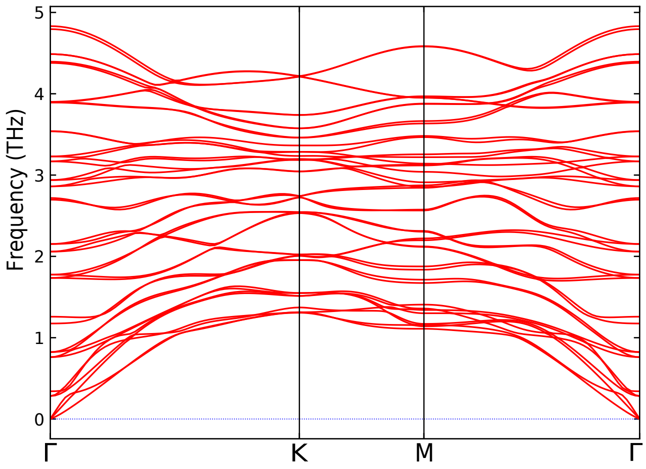


Figure S14: Phonon spectrum along a high-symmetry path in the Brillouin zone.

## 7 Magnetic fluctuations in paramagnetic phase

**Quasielastic scattering** In this section, we discuss quasielastic scattering (QES) in paramagnetic phase. We first apply Bose factor correction to obtain normalized Raman susceptibility:

$$\frac{\chi''(\omega)}{\omega} = \frac{I(\omega)}{(n+1)\omega} \tag{27}$$

where $n$ is the Bose-Einstein factor and $I(\omega)$ is the Raman intensity. We observe clear background changes as a function of the layer thickness (Fig. S15). We attribute this background to quasielastic scattering for magnetic fluctuations. The spin fluctuations couple the light to the magnetic energy, giving rise to the peak at zero Raman frequency shift, the linewidth of spin-lattice relaxation time, and the intensity that is proportional to magnetic specific heat. We extract the quasielastic scattering using a magnetic fluctuation model,

$$\frac{\chi''(\omega)}{\omega} \propto C_{\mathrm{m}} T \frac{Dk^2}{\omega^2 + (Dk^2)^2} \tag{28}$$

where $C_m$ is magnetic specific heat and $D$ is the magnetic contribution to thermal conductivity [33–35]. The quasielastic scattering is enhanced in thinner layers. This illustrates that the magnetic fluctuations are stabilized as the magnetic system approaches to bulk regime. Moreover, the spin fluctuations involve the relaxation channel by coupling with lattice vibrations [33,36]. The spin-lattice coupling was observed in our previous study.

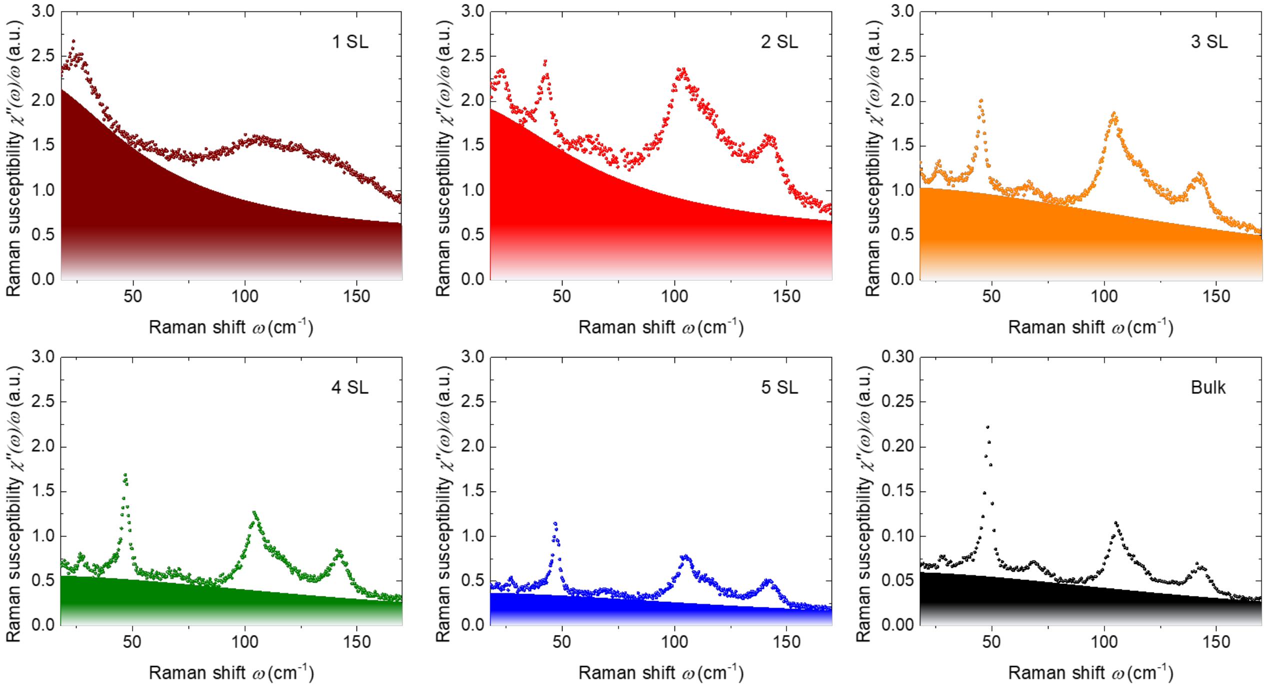


Figure S15: Layer-dependent quasielastic scattering in paramagnetic phase. The colored shades represent the extracted quasielastic scattering from magnetic fluctuation model.

**Dynamic Raman susceptibility** To compare magnetic fluctuations quantitatively, we extract the dynamic Raman susceptibility by integrating the normalized Raman susceptibility up to $180\ cm^{-1}$ using the Kramers-Kronig relations as follows.

$$\chi''_{dyn} = \frac{2}{\pi}\int_0^{\Omega}\frac{\chi''}{\omega}dx \tag{29}$$

## 8 Temperature dependent dynamic Raman susceptibility

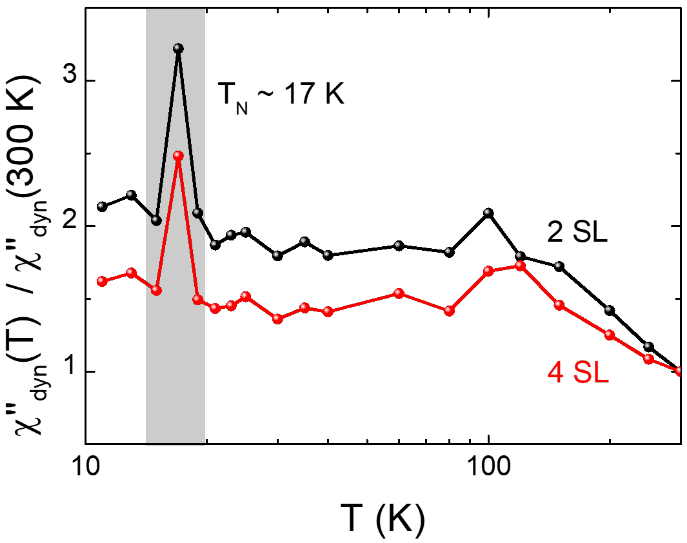


Figure S16: Temperature dependence of dynamic Raman susceptibility for 2 and 3 SL. The grey shade indicates the critical behavior across the Nèel temperature.

We analyze temperature dependence of dynamic Raman susceptibility $\chi''_{dyn}$ in 2 and 3 SLs (Supplementary Figure S17). We observe $\chi''_{dyn}$ is peaked at the Nèel temperature, $\sim$ 17 K. The

observed Nèel temperature for 2 SLs is consistent with the integrated intensity analysis shown in Fig. 2c. The difference between 2 and 3 SLs is unclear due to the limited temperature step (2 K). $\chi''_{dyn}$ is proportional to magnetic specific heat $C_m$, which shows the critical behavior of magnetic phase transition [34,35]. This temperature dependence support our analysis of the QES as evidence for increasing magnetic fluctuations with decreasing layer thickness.

## 9 Layer dependent low frequency phonon spectra

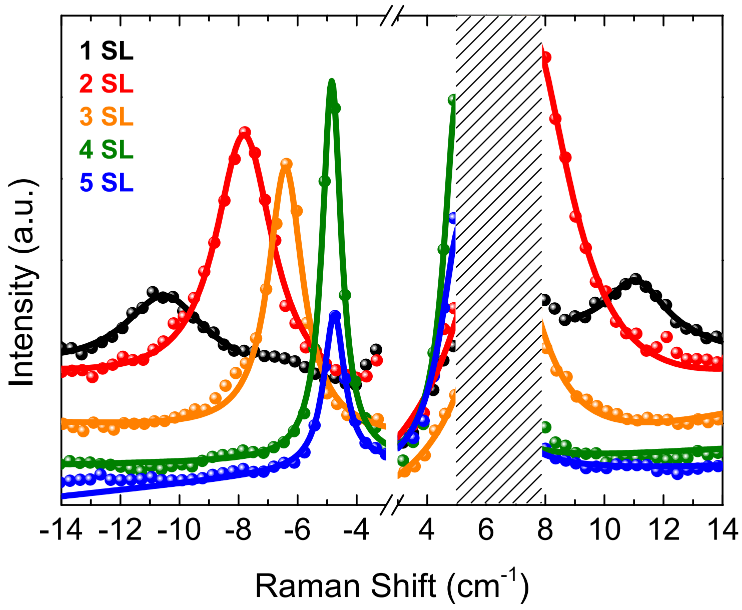


Figure S17: Layer dependence of low frequency Raman spectra in 1 to 5 SLs. The data is taken at room temperature. The stripe-pattered shade is to block the noise line. The solid lines are Lorentzian model fits.

Low-frequency phonon spectra taken at room temperature using collinearly polarized incident and scattered light. The phonon mode corresponds to a breathing mode. The systematic frequency shift as a function of layer thickness can be modeled by a linear chain model. The mode observed from the 1-SL corresponds to lattice vibration against the substrate. The presence of this mode suggest the crystalline structure of 1-SL is retained.

1. Li, B. *et al.* Competing magnetic interactions in the antiferromagnetic topological insulator $\mathrm{mnbi_2te_4}$. *Phys. Rev. Lett.* **124**, 167204 (2020). URL `https://link.aps.org/doi/10.1103/PhysRevLett.124.167204`.

2. Li, B. *et al.* Two-dimensional ferromagnetism with long-range interactions in the layered magnetic topological insulator mnbi2te4 (2020). URL `https://arxiv.org/abs/2007.08468`.

3. Yang, S. *et al.* Odd-even layer-number effect and layer-dependent magnetic phase diagrams in $\mathrm{mnbi_2te_4}$. *Phys. Rev. X* **11**, 011003 (2021). URL `https://link.aps.org/doi/10.1103/PhysRevX.11.011003`.

4. Ovchinnikov, D. *et al.* Intertwined topological and magnetic orders in atomically thin chern insulator mnbi2te4. *Nano Letters* **21**, 2544–2550 (2021). URL `https://doi.org/10.1021/acs.nanolett.0c05117`.

5. Rezende, S. M., Azevedo, A. & Rodríguez-Suárez, R. L. Introduction to antiferromagnetic magnons. *Journal of Applied Physics* **126**, 151101 (2019). URL `https://doi.org/10.1063/1.5109132`. `https://doi.org/10.1063/1.5109132`.

6. Stokes, H. T., Hatch, D. M. & Campbell, B. J. URL `https://stokes.byu.edu/iso/findsym.php`.

7. Stokes, H. T. & Hatch, D. M. Findsym: Program for identifying the space group symmetry of a crystal. *J. Appl. Cryst.* **28**, 237–238 (2005).

8. Bilbao crystallographic server. https://www.cryst.ehu.es/. Accessed: 2021-01-07.

9. Rodriguez-Vega, M., Leonardo, A. & Fiete, G. A. Group theory study of the vibrational modes and magnetic order in the topological antiferromagnet $MnBi_2Te_4$. *Phys. Rev. B* **102**, 104102 (2020). URL https://link.aps.org/doi/10.1103/PhysRevB.102.104102.

10. Xu, Y. *et al.* High-throughput calculations of magnetic topological materials. *Nature* **586**, 702–707 (2020). URL https://doi.org/10.1038/s41586-020-2837-0.

11. Elcoro, L. *et al.* Magnetic topological quantum chemistry (2020). 2010.00598.

12. Bilbao crystallographic server, corepresentations of magnetic point groups. https://www.cryst.ehu.es/cryst/mpointrepres.html. Accessed: 05-26-2021.

13. Anastassakis, E. & Burstein, E. Morphic effects. v. time reversal symmetry and the mode properties of long wavelength optical phonons. *Journal of Physics C: Solid State Physics* **5**, 2468–2481 (1972). URL https://doi.org/10.1088/0022-3719/5/17/021.

14. Cracknell, A. P. Scattering matrices for the raman effect in magnetic crystals. *Journal of Physics C: Solid State Physics* **2**, 500–511 (1969). URL

15. YU, P. & Cardona, M. *Fundamentals of Semiconductors: Physics and Materials Properties*. Graduate Texts in Physics (Springer Berlin Heidelberg, 2010). URL https://books.google.com/books?id=5aBuKYBT_hsC.

16. Daniel, M. R. & Cracknell, A. P. Magnetic symmetry and antiferromagnetic resonance in coo. *Phys. Rev.* **177**, 932–941 (1969). URL https://link.aps.org/doi/10.1103/PhysRev.177.932.

17. Nguyen, T. M. H. *et al.* Two-magnon scattering in the 5d all-in-all-out pyrochlore magnet cd2os2o7. *Nature Communications* **8**, 251 (2017). URL https://doi.org/10.1038/s41467-017-00228-w.

18. Chinn, S. R., Zeiger, H. J. & O'Connor, J. R. Two-magnon raman scattering and exchange interactions in antiferromagnetic $knif_3$ and $k_2nif_4$ and ferrimagnetic $rbnif_3$. *Phys. Rev. B* **3**, 1709–1735 (1971). URL https://link.aps.org/doi/10.1103/PhysRevB.3.1709.

19. Gretarsson, H. *et al.* Two-magnon raman scattering and pseudospin-lattice interactions in $sr_2iro_4$ and $sr_3ir_2o_7$. *Phys. Rev. Lett.* **116**, 136401 (2016). URL https://link.aps.org/doi/10.1103/PhysRevLett.116.136401.

20. Rigitano, D., Vaknin, D., Barberis, G. E. & Granado, E. Raman scattering from one and two magnons in magnetoelectric $linipo_4$. *Phys. Rev. B* **101**, 024417 (2020). URL https://link.aps.org/doi/10.1103/PhysRevB.101.024417.

21. Fleury, P. A. & Loudon, R. Scattering of light by one- and two-magnon excitations. *Phys. Rev.* **166**, 514–530 (1968). URL https://link.aps.org/doi/10.1103/PhysRev.166.514.

22. Chen, C.-C., Jia, C. J., Kemper, A. F., Singh, R. R. P. & Devereaux, T. P. Theory of two-magnon raman scattering in iron pnic-

tides and chalcogenides. *Phys. Rev. Lett.* **106**, 067002 (2011). URL https://link.aps.org/doi/10.1103/PhysRevLett.106.067002.

23. Li, S. *et al.* Symmetry-resolved two-magnon excitations in a strong spin-orbit-coupled bilayer antiferromagnet. *Phys. Rev. Lett.* **125**, 087202 (2020). URL https://link.aps.org/doi/10.1103/PhysRevLett.125.087202.

24. Das, S. K., Singh, V. N. & Majumdar, P. Magnon spectrum in the domain ferromagnetic state of antisite-disordered double perovskites. *Phys. Rev. B* **88**, 214428 (2013). URL https://link.aps.org/doi/10.1103/PhysRevB.88.214428.

25. Calderon Filho, C., Gomes, P., García-Flores, A., Barberis, G. & Granado, E. Two-magnon raman scattering in limnpo4. *Journal of Magnetism and Magnetic Materials* **377**, 430–435 (2015). URL https://www.sciencedirect.com/science/article/pii/S0304885314010130.

26. Toft-Petersen, R. *et al.* Magnetic phase diagram of magnetoelectric limnpo$_4$. *Phys. Rev. B* **85**, 224415 (2012). URL https://link.aps.org/doi/10.1103/PhysRevB.85.224415.

27. *Dynamical properties of Solids*, vol. 4 of *Disordered Solids, Optical properties* (North-Holland Publishing Company, 1980).

28. Kresse, G. & Furthmüller, J. Efficiency of ab-initio total energy calculations for metals and semiconductors using a plane-wave ba-

sis set. *Computational Materials Science* **6**, 15–50 (1996). URL http://www.sciencedirect.com/science/article/pii/0927025696000080.

29. Kresse, G. & Furthmüller, J. Efficient iterative schemes for ab initio total-energy calculations using a plane-wave basis set. *Phys. Rev. B* **54**, 11169–11186 (1996). URL https://link.aps.org/doi/10.1103/PhysRevB.54.11169.

30. Grimme, S., Antony, J., Ehrlich, S. & Krieg, H. A consistent and accurate ab initio parametrization of density functional dispersion correction (dft-d) for the 94 elements h-pu. *J. Chem. Phys.* **132**, 154104 (2010).

31. Otrokov, M. M. *et al.* Prediction and observation of an antiferromagnetic topological insulator. *Nature* **576**, 416–422 (2019). URL http://www.nature.com/articles/s41586-019-1840-9.

32. Togo, A. & Tanaka, I. First principles phonon calculations in materials science. *Scripta Materialia* **108**, 1–5 (2015). URL https://www.sciencedirect.com/science/article/pii/S1359646215003127.

33. Halley, J. W. Light scattering as a probe of dynamical critical properties of antiferromagnets. *Phys. Rev. Lett.* **41**, 1605–1608 (1978). URL https://link.aps.org/doi/10.1103/PhysRevLett.41.1605.

34. Reiter, G. F. Light scattering from energy fluctuations in magnetic insulators. *Phys. Rev. B* **13**, 169–173 (1976). URL https://link.aps.org/doi/10.1103/PhysRevB.13.169.

35. Kim, K. *et al.* Suppression of magnetic ordering in XXZ-type antiferromagnetic monolayer NiPS3. *Nat. Commun.* **10**, 345 (2019). URL http://www.nature.com/articles/s41467-018-08284-6.

36. Lemmens, P., Güntherodt, G. & Gros, C. Magnetic light scattering in low-dimensional quantum spin systems. *Phys. Rep.* **375**, 1–103 (2003). URL https://www.sciencedirect.com/science/article/pii/S0370157302003216.
